# Ambiguity is a linking feature for interocular grouping

**DOI:** 10.1167/jov.22.11.12

**Published:** 2022-10-20

**Authors:** Sunny M. Lee, Emily Slezak, Steven K. Shevell

**Affiliations:** 1 Department of Psychology and Institute for Mind and Biology, University of Chicago, Chicago, IL, USA; 2 Department of Psychology and Institute for Mind and Biology, University of Chicago, Chicago, IL, USA; 3 Department of Psychology and Institute for Mind and Biology, University of Chicago, Chicago, IL, USA; 4 Department of Ophthalmology & Visual Science, University of Chicago, Chicago, IL, USA

**Keywords:** neural ambiguity, color perception, grouping, perceptual resolution, interocular-switch rivalry

## Abstract

Ambiguity is implicit in neural representations of the physical world. Previous work has examined how the visual system resolves ambiguous neural signals that represent various features, such as the percept resulting from rivalrous chromaticities or forms. Relatively little is known, however, about the contribution of *un*ambiguous neural representations to perceptual resolution of ambiguous ones. This is addressed here by measuring perceptual resolution of ambiguity by grouping, which is operationalized as the tendency for multiple similar ambiguous representations to be seen as identical to each other. Multiple chromatically ambiguous representations were created using interocular switch rivalry and presented together with a nearby but separate unambiguous (non-rivalrous) chromaticity. The magnitude of grouping the chromatic regions was compared when ambiguous regions were seen alone versus with unambiguous regions seen simultaneously. Contrary to prevailing theory that the resolution of the ambiguous percepts would follow the unambiguous ones, the ambiguous chromatic regions consistently appeared identical to each other, but their appearance was not found to be attracted to the unambiguous color percept. This supports the proposition that the ambiguity itself in a neural representation is a linking feature contributing to perceptual disambiguation.

## Introduction

Ambiguity is inherent in visual neural representations. One cause is that the biological representations of the three-dimensional world are generated from two-dimensional projections on the surface of each retina (the inverse optics problem) (Palmer, 1999). Ambiguity results also from neural processes that extrapolate incomplete low-level representations so we perceive stable recognizable objects and, further, often from unequal left-eye and right-eye neural signals that compete for perceptual dominance at a particular location in the visual field. Thus, in natural viewing our percepts of the fundamental features of an object, such as color, form, and motion, often follow from ambiguous information from the retinal images of the eyes. These representations are resolved so that we experience stable coherent percepts (Alais & Blake, 2015; for a review, see Brascamp & Shevell, 2021). Remarkably, perceptual resolution of ambiguity usually is effortless and unnoticed in our visual experience of a rich three-dimensional environment.

**Figure 1.**
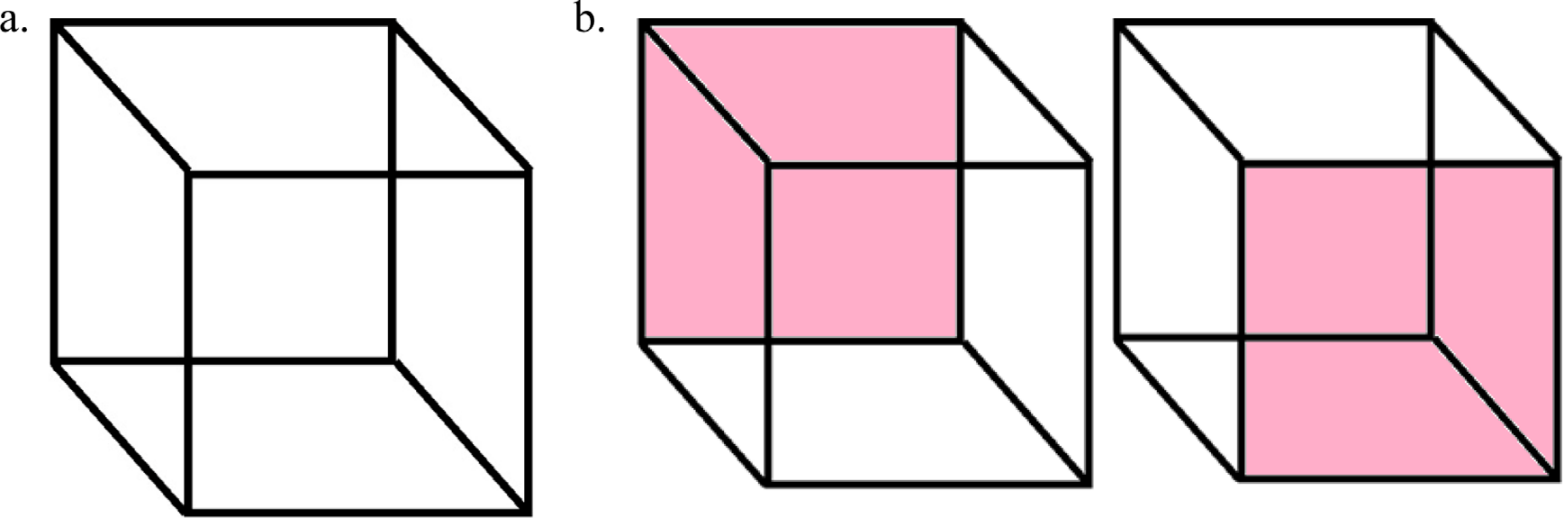
(a) A Necker cube is an example of a figure producing bistable percepts. One interpretation is immediately elicited, but after a few seconds it can spontaneously switch to a different percept. (b) Two possible interpretations of a Necker cube that depend on which of the highlighted faces is seen as the front face of the cube (either the upper-left or lower-right square).

Under certain circumstances a given retinal image fails to lead to a single percept; instead, what an observer sees varies over time with two or more different percepts seen one after the other (Walker, 1975; Leopold & Logothetis, 1999). This multistability of percepts is evoked by the well-known Necker cube (Necker, 1832) (Figure 1a) and Rubin's vase (Rubin, 1921; Eagleman, 2001), which makes salient the competition that accompanies perceptual resolution of neural representations. Although multistable percepts are rare, the perceptual stability and fluidity that we experience in normal viewing are a product of neural processes that extrapolate and disambiguate relatively sparse and often rapidly changing retinal signals (Tong, Meng, & Blake, 2006).

How can ambiguous neural signals be resolved? Supplemental context can be an important factor. Context may be from a different part of the visual field, an earlier moment in time, another one of our senses, or even prior knowledge (Brascamp & Shevell, 2021). This study focuses on simultaneously viewed context in separate retinotopic areas. Here, the context may be chosen to have *un*ambiguous features in order to determine whether perceptual resolution of ambiguous neural representations is attracted to the percepts from nearby unambiguous representations. The percept studied here, as a model system, is color.

When multiple, identical ambiguous representations are in separate parts of the visual field these representations have potential to be their own context. Consider a case in which every representation is driven by identical competing stimuli. An example for color is competition for visual dominance between rivalrous chromaticities that appear red or green. When all ambiguous representations in various parts of the visual field are identical, the multiple representations may become linked with each other by virtue of their identical, though competing, neural responses. This link may cause all of the multiple percepts to be seen as identical to each other in color (either all of them seen as red or all as green) even without specifying which color is perceived at a particular moment. In fact, this occurs with multiple simultaneous and identical ambiguous representations: All objects often are seen as the same color even though their appearance varies over time—in this case, from all red to all green. Thus, perceptual resolution of multiple ambiguous neural representations leads to seeing all of them as identical far more than if each representation is resolved independently (Kovács, Papathomas, Yang & Fehér, 1996; Dobbins & Grossman, 2018; Shevell, 2019).

Here, experiments consider the role of context composed of both multiple identical ambiguous representations and also nearby unambiguous representations to determine whether the resolution of ambiguous signals is altered by the unambiguous ones. A possible effect of unambiguous context that appears, say, red is to attract ambiguous representations to the same percept. Attraction to an unambiguous object with similar features has been investigated mostly using moving or three-dimensional stimuli. The perceived rotational direction of an ambiguous structure-from-motion object, when presented with an object having unambiguous rotation, tends to be in the same direction as the unambiguous one (Freeman & Driver, 2006). The coupling between ambiguous and unambiguous motion breaks down, however, if the objects are not plausibly part of a single larger motion context. This can occur if the two objects are not rotating coaxially (Freeman & Driver, 2006) or if the two objects are rotating cylinders that appear less likely to be parts of a single partially occluded object due to not sharing a far depth plane (Klink, Noest, Holten, van den Berg, & van Wezel, 2009). Similarly, using binocular rivalry instead of structure-from-motion to present ambiguous and unambiguous motion together, ambiguous motion tends to resolve identically to the unambiguous motion but only if the parts form a coherent global figure (Sobel & Blake, 2002). Note the confound of motion and form in these experiments, which limits a conclusion about the general influence of unambiguous motion on ambiguous motion.

Studies using Necker cubes presented together with unambiguously oriented cubes also show that resolution of the orientation of the Necker cube tends to follow the nearby unambiguous cubes (Sundareswara & Schrater, 2008; Ouhnana & Kingdom, 2016), but these studies are similarly limited by the confound of three-dimensional plausibility. The measurements do not separate the direct influence of nearby unambiguous stimuli from the tendency to see all of the presented stimuli together as parts of a single plausible three-dimensional interpretation (e.g., a viewed-from-above percept of a Necker cube may not be plausible, and therefore suppressed, because it would require a different viewpoint than for nearby unambiguous context that appears to be viewed from below). This confound limits the relevance of earlier work with Necker cubes or motion stimuli for answering the general question of how unambiguous context affects perceptual resolution of ambiguity.

A different process by which context may resolve ambiguity is linking of ambiguous representations according to their common (even if ambiguous) features such as color, orientation, or motion. This extends Barlow's (1981) linking-features hypothesis to features that compete for conscious awareness. Common ambiguity shared by multiple neural representations may cause them to appear identical. If common ambiguity among objects mediates their perceptual resolution, then unambiguous and ambiguous representations may not be linked with each other to appear the same, implying little or no effect of unambiguous representations on perceptual resolution of ambiguous ones.

This view is supported by studies with stimuli having more than one feature. They show that feature conjunction in one retinal area can dominate the perceived conjunction of the same features in another area. For example, a central region with overlapping red dots moving upward and green dots moving downward, presented simultaneously with peripheral red and green dots moving in opposite directions (red dots downward and green upward), often gives the percept of all the red dots moving upward and all the green dots downward (Wu, Kanai, & Shimojo, 2004; Wang & Shevell, 2014; Shevell & Wang, 2016). Thus, the red and green dots have a veridical perceived direction of motion in the central visual field but not in the periphery. The perceived color–motion conjunctions in the periphery follow the central–field conjunctions when all stimuli have, and presumably are linked by, identical size, density, speed, directions of motion, and chromaticities.

Multiple rivalrous dichoptic stimuli also are linked by their stimulus features in interocular grouping (Kovács et al., 1996). For example, an array of 24 binocularly rivalrous discs at chromaticities normally seen as green and red can be presented with 12 of the discs in each eye at one chromaticity and the other 12 at the other chromaticity (patchwork rivalry) (Kovács et al., 1996). Each disc in one eye has a retinotopically corresponding disc in the other eye with the rivalrous chromaticity. Despite the absence of a monocular stimulus composed of all discs at one chromaticity, observers often see all 24 discs as red and then, subsequently, all 24 discs as green. A more recent study, with 16 rather than 24 chromatic discs, found that all 16 were perceived to be the same color for nearly 50% of the viewing time by most observers (Slezak & Shevell, 2018), a strikingly large proportion of the time compared with the chance level of 1% assuming that the color of each disc had an independent 50–50 chance of visual dominance at each moment. This result is consistent with a coherent percept composed of all discs seen as the same color due to linking the discs by their common features (their rivalrous chromaticities) at a neural level that incorporates responses from both eyes.

Further evidence for linking by common ambiguity is that equally ambiguous stimuli tend to be perceived identically more often than stimuli with different degrees of ambiguity (Ramachandran & Anstis, 1985). Moreover, reducing the similarity of ambiguity has a substantial effect. When two moving ambiguous objects such as kinetic dot cubes are manipulated independently to alter the transparency of perspective information (thereby differentially biasing perception of each object in favor of one of two possible directions of rotation), the objects are perceived to be rotating in the same direction up to 30% less often than when both objects have the same transparency and thus are equally ambiguous (Grossmann & Dobbins, 2003).

In sum, ambiguity is pervasive in visual neural representations (Brascamp & Shevell, 2021), and its resolution is a fundamental task of the visual system. Experiments here test whether the visual system resolves the ambiguity in one retinotopic area by using, or not, unambiguous representations in separate retinotopic regions. The experiments address also (a) whether ambiguity itself can be considered a linking feature for multiple stimuli in view simultaneously so that common ambiguity in separate retinotopic areas causes them to appear the same; (b) whether linking by ambiguity can occur even when an alternate cue from unambiguous features is available; and (c) whether disambiguation of a neural representation occurs before linking separate regions, rather than linking by the *ambiguity itself* prior to perceptual resolution.

## Methods

### Apparatus

**Figure 2.**
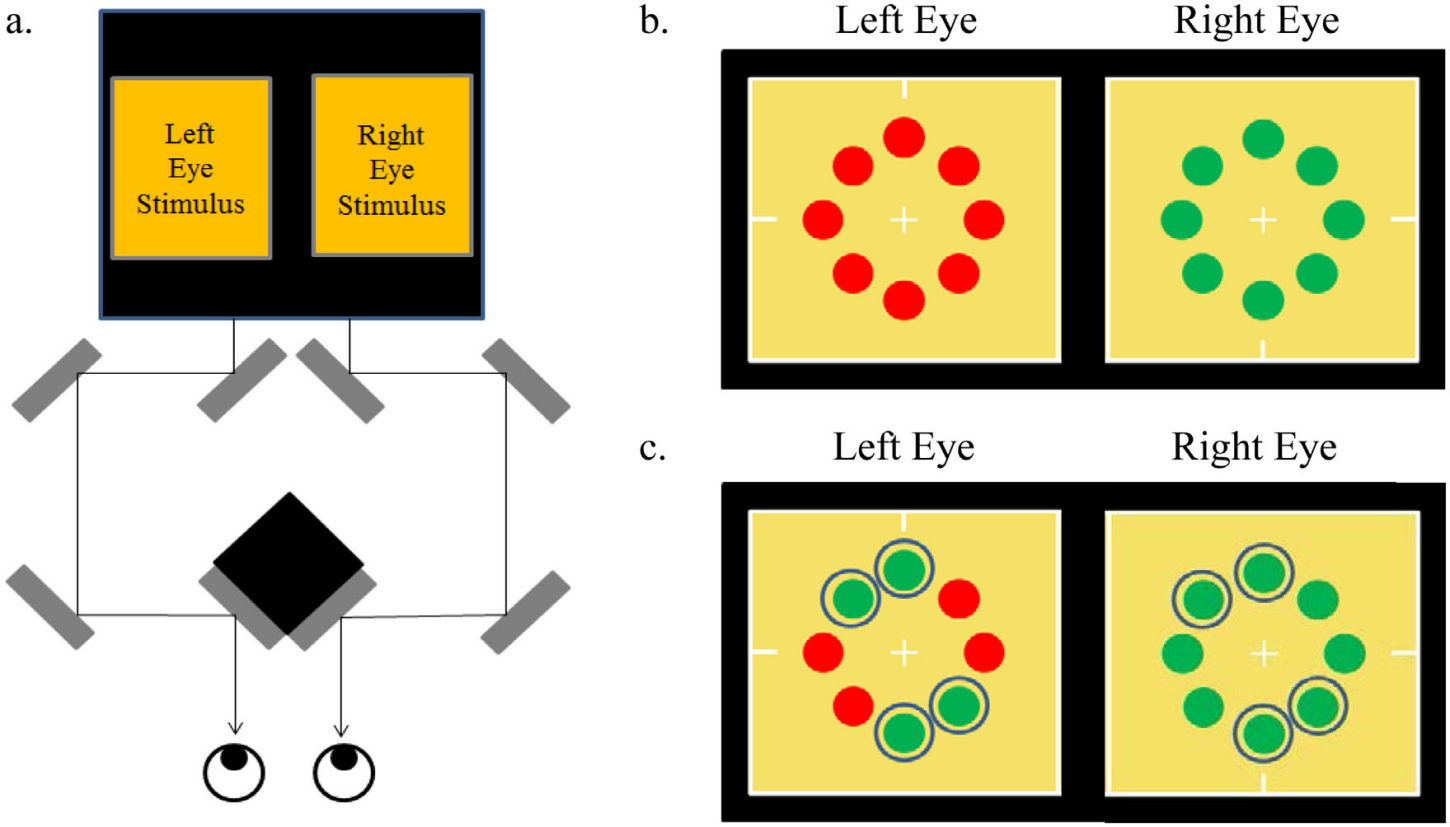
Stimulus presentation and arrangement. (a) Haploscope used in the experiments composed of eight mirrors (gray bars) positioned to present different stimuli to each eye (arrows) at an optical-path distance of 115 cm. (b) All-ambiguous condition in which all discs were presented using CISR (the left eye and right eye images were swapped at 3.75 Hz). (c) Mixed-ambiguity condition in which ambiguous and unambiguous discs were presented simultaneously. Unambiguous discs are circled here for clarity but the circles were not part of the actual stimulus display.

In all experiments, observers viewed a calibrated computer monitor (Sony GDM-F520 CRT Display, 1360×1024-pixel resolution, 75-Hz refresh rate; Sony Group, Tokyo, Japan) controlled by an iMac computer (Apple Inc., Cupertino, CA) running software developed with Xcode. The monitor was viewed through a haploscope at an optical-path distance of 115 cm and in a dark room (Figure 2a). Observers used a chinrest to center their gaze and minimize head movement.

### Stimuli

The experiments used chromatic interocular-switch rivalry (CISR) (Christiansen, D'Antona, & Shevell, 2017), a form of stimulus rivalry. In contrast to standard binocular rivalry with rivaling monocular images presented steadily to each eye, CISR presents rivaling chromatic stimuli to each eye and swaps them between eyes several times a second. CISR aims to strip out eye-of-origin information (Slezak & Shevell, 2018), which often causes observers to perceive only stimuli presented to one eye (Stuit, Paffen, van der Smagt, & Verstraten, 2011). As in standard binocular rivalry, CISR results in slow alternations between bistable percepts—in this case, two colors. CISR is used because stimuli presented using standard binocular rivalry confound ambiguity of the percept with eye of origin of the stimulus, thus constraining how ambiguity can be experimentally measured and manipulated.

The haploscopic display allowed presentation of a different image to each eye at each corresponding retinal location. The right-eye stimulus had discs in exact retinal correspondence with discs in the left eye but at a different chromaticity in order to produce rivalry. A total of eight discs could be presented, all equidistant from the central fixation cross (Figures 2b and 2c).

The disc at each retinotopic location was one of two chromaticities (MacLeod & Boynton, 1979): *L*/(*L* + *M*) or *S*/(*L* + *M*) of (0.72, 0.30), called “red,” or (0.61, 0.30), called “green.” The background had chromaticity (0.665, 0.30), an intermediate point between the two disc chromaticities. The luminance of the display was 5 cd/m^2^. Discs were 0.5° in diameter with centers 1° away from fixation. In various experimental conditions, the disc at each retinotopic location was presented either ambiguously using CISR or unambiguously with the same chromaticity presented to both eyes. Experiments were comprised mainly of two types of stimulus arrangements: “all-ambiguous” with all presented discs in rivalry (e.g., Figure 2b) or “mixed-ambiguity” with one or more discs not rivalrous (Figure 2c). Specific stimuli are described below for each experiment.

Ambiguous discs in CISR were exchanged between eyes at a frequency of 3.75 Hz, or 7.5 times a second, such that a disc in one eye was “red” when the disc in the corresponding location of the other eye was “green.” The unambiguous non-rivalrous discs changed chromaticities simultaneously in both eyes at 0.25 Hz, or once every 2 seconds. The unambiguous discs switched chromaticities for two purposes: to prevent chromatic adaptation to a single chromaticity and to measure each possible color percept (so that all discs could be seen as “green” or all discs as “red”). This frequency was set low so that the perceived duration of each color percept could be easily measured. In addition to the discs around the fixation cross, the stimulus included a surrounding white square with Nonius lines (small lines protruding inward from the surrounding white square; see Figures 2b and 2c). The white square encompassing the discs aided stable fixation for the rivalrous discs. Nonius lines in the left-eye image were at the top and left and in the right-eye the bottom and right. Observers when properly fused perceived Nonius lines aligned horizontally (the left and right lines) and vertically (the top and bottom lines).

### Procedure

Before the experiments began, heterochromatic flicker photometry was performed by each observer to individually determine equiluminant settings for stimulus presentation (Christiansen, D'Antona, & Shevell, 2009). This was repeated on at least three separate days to check the stability of the measurements. The personalized displays ensured that the “red” and “green” chromaticities presented to observers were equiluminant, preventing any confound from luminance contrast.

Observers took part in one practice session and then three experimental sessions, each on a different day, to assess intra-observer variability. Conditions were run in a random order. Four trials blocked together for each condition were run in every session. Between blocks, observers were given a break to limit fatigue.

The observers were instructed to report via a game-pad button when they perceived *all* of the discs in view as the same color, either all “red” or all “green,” holding down a response button for the entire duration they perceived all the discs as one of the colors. Each trial lasted 70 seconds, but the first 10 seconds were not recorded due to possible differences in adaptation between the two eyes from the onset chromaticities. The proportion of time an observer saw all discs of the same color (either all “green” or all “red”) out of 60 seconds was used as the measure of total dominance time for each percept. Following well-established nomenclature (Kovács et al., 1996; Stuit et al., 2011; Dobbins & Grossman, 2018), perceptual grouping was operationalized using measures of total dominance time. Of course, temporal coherency is not the only example of grouping (Dobbins & Grossman, 2018; Herzog, 2018).

### Observers

All observers except author SL were naïve about the purpose and design of the experiments. Written consent in accordance with the policy of the University of Chicago's Institutional Review Board was obtained from each observer. Prior to participating, Ishihara plates and an anomaloscope for Rayleigh matching were used to screen for observers with normal color vision. The Titmus stereo test was administered to check stereoscopic vision. All observers had normal (or corrected-to-normal) visual acuity, color vision, and stereoscopic vision. One observer was excluded during screening due to failing the Titmus stereo test based on a minimum criterion of six of nine correct responses on the graded circle test and perceiving the fly wings above the plate in the stereo fly test.

## Results

### Experiment 1: Is perceptual resolution of ambiguous chromatic neural representations affected by unambiguous representations?

Experiment 1 determined whether ambiguity itself could contribute to grouping multiple objects. Grouping was assessed using the proportion of time that all discs, ambiguous or unambiguous, were simultaneously perceived as the same color. Multiple separate retinotopic areas with ambiguous chromatic representations are known to resolve together perceptually to all appear the same color more often than chance (Slezak & Shevell, 2018; Shevell, 2019). Experiment 1 tested how unambiguous color percepts affected the perceptual resolution of the ambiguous ones by comparing the dominance time of perceiving as identical ambiguous stimuli presented alone versus ambiguous and unambiguous stimuli presented simultaneously.

**Figure 3.**
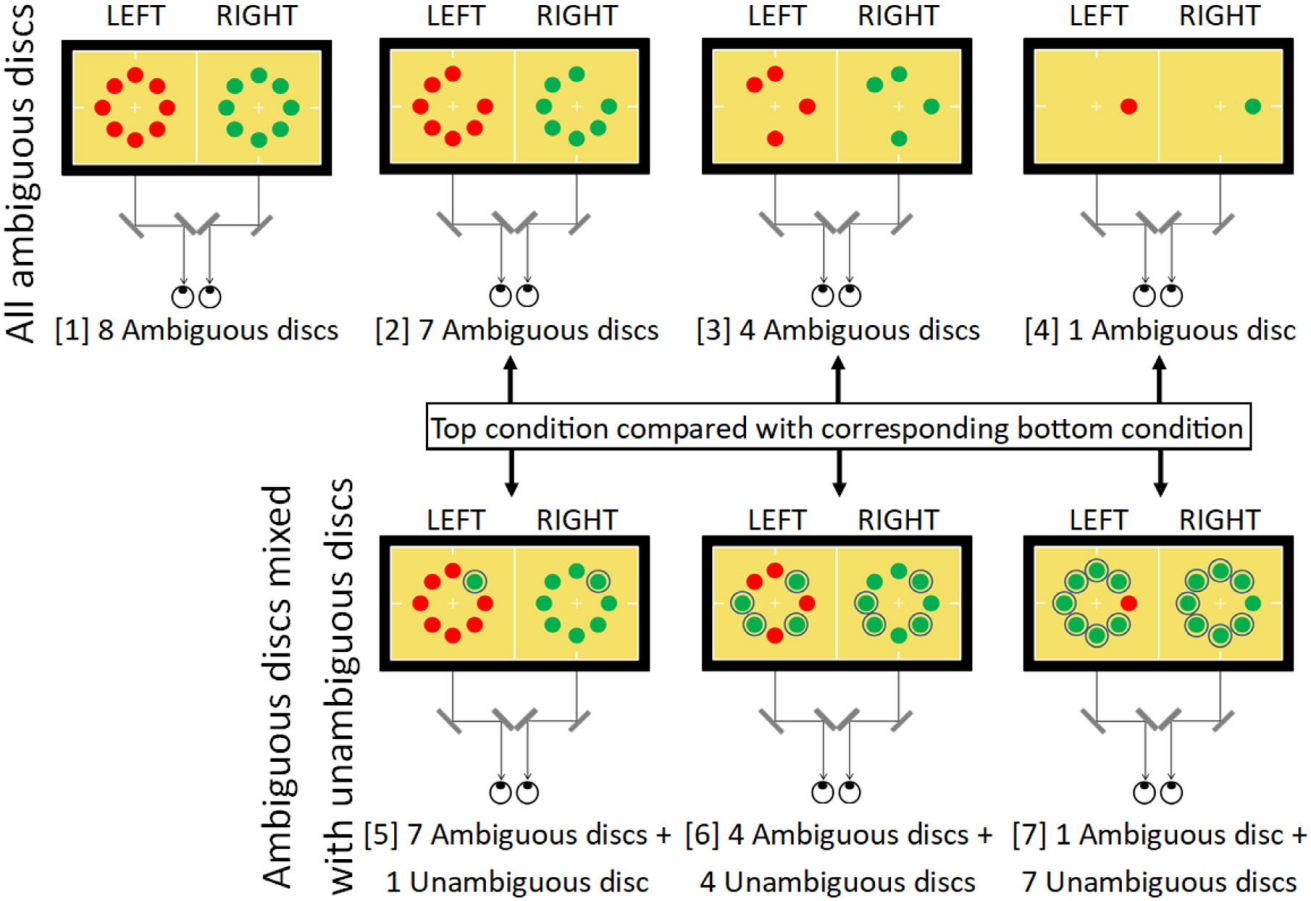
Stimuli in Experiment 1. All-ambiguous conditions (top row) were compared with the corresponding mixed-ambiguity condition immediately below. Unambiguous discs are circled for clarity in the figure but the circles were not used in the experiments. Positions of ambiguous and unambiguous discs were randomized on every trial. The proportion of time observers saw all discs as the same color was measured.

In this experiment, seven different stimulus displays varied the number of ambiguous discs presented with CISR and also the number of unambiguous (non-rivalrous) discs presented simultaneously. Four conditions had all ambiguous discs in different numbers: eight, seven, four, or one ambiguous disc(s) (conditions 1, 2, 3, and 4, respectively, as shown in Figure 3). Three conditions had mixed ambiguity: seven ambiguous discs with one unambiguous disc, four ambiguous discs with four unambiguous discs, or one ambiguous disc with seven unambiguous discs (conditions 5, 6, and 7, respectively). Condition 2 with seven ambiguous discs was compared with the mixed-ambiguity condition 5 with seven ambiguous discs and one unambiguous disc. Similar comparisons were condition 3 versus 6 with four ambiguous discs, and condition 4 versus 7 with one ambiguous disc. Condition 1, composed of eight ambiguous discs, was a control for the effect of reducing the total number of discs displayed in condition 2 from eight to seven. In sum, these comparisons tested whether grouping among only ambiguous discs of a given number (seven, four, or one) was greater than grouping among the same ambiguous discs seen also with an unambiguous contextual color cue from non-rivalrous discs.

Five observers were tested and analyzed separately. For each condition, the proportions of time out of 60 seconds that observers saw all “red” discs or all “green” discs were added together to give a total proportion of time observers saw a stable percept of a single color. The proportions were arcsine transformed prior to analysis in order to better approximate a normal distribution (Kirk, 2013).

**Figure 4.**
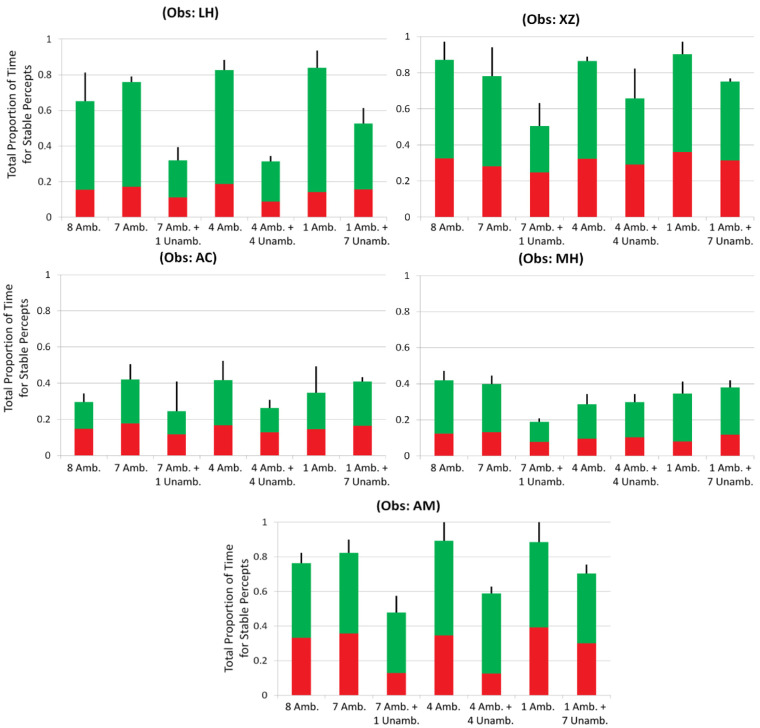
Experiment 1 results. Comparison of all-ambiguous versus mixed-ambiguity conditions for varying numbers of ambiguous and unambiguous discs. The red portion of each bar shows the proportion of time seeing all red discs, and the stacked green bar the proportion of time for all green discs. The overall height of a bar is the proportion of time seeing all discs of the same color.

Measurements for each of the seven conditions (Figure 4) are shown separately for each observer. A two-way analysis of variance (ANOVA) was conducted, with factor 1 being all-ambiguous conditions against mixed-ambiguity conditions; factor 2 was the number of ambiguous discs displayed. For this analysis, condition 1 with eight ambiguous discs was excluded, as it did not have a mixed-ambiguity counterpart. Four of the five observers (all except MH) showed a significant difference between the two levels of factor 1 with higher dominance time for the all-ambiguous conditions than the mixed-ambiguity conditions: *p* < 0.05 for each of the four observers, and *F*(1, 12) > 4.75 in every case. The measurement for MH was in the same direction but did not reach significance. Two of the five observers (LH and XZ) also showed a small though significant difference for factor 2, signifying that the number of ambiguous discs present affected their dominance times: *p* < 0.05, and *F*(2, 12) > 3.89 for both. This may reflect the unique feature of the conditions with a single ambiguous disc. For both of these observers, the greatest dominance time was for a single ambiguous disc, for which no linking with other ambiguous stimuli was necessary (this held for both their all-ambiguous and mixed-ambiguity conditions). Also, a one-way ANOVA was performed to test for a difference due to the number of ambiguous discs presented in only the all-ambiguous conditions, including the control condition 1 with eight ambiguous discs. There was never a significant difference for any of the observers: *p* > 0.1, and *F*(3, 8) < 2.92 for every observer.

In a further analysis that combined results across observers, two specific features of the measurements directly addressed whether ambiguous discs were linked with unambiguous discs. First, for each of the five observers, consider three direct comparisons of whether a given number of ambiguous discs (seven, four, or one) was attracted to the color of unambiguous discs (one, four, or seven of them, respectively; conditions 2 vs. 5, 3 vs. 6, and 4 vs. 7). With three comparisons for each of five observers there were 15 comparisons in all, and in 12 of these 15 cases adding unambiguous disc(s) reduced, rather than increased, the proportion of time seeing all discs of the same color (*p* < 0.04 by a two-tailed binomial test, with chance probability *p* = 0.5). Second, compare condition 2 with seven ambiguous discs to any condition that included unambiguous discs (conditions 5, 6, or 7); for every observer, the largest proportion of time seeing all discs of the same color was the condition with only the seven ambiguous discs (*p* < 0.001 by a binomial test for five observers, each with a chance probability *p* = 0.25). The same conclusion held comparing condition 1 with eight ambiguous discs to the conditions with unambiguous discs (conditions 5, 6, or 7). In this case, the largest proportion of these four conditions was condition 1 for four of the five observers (*p* < 0.02, again by a binomial test for five observers, each with a chance probability *p* = 0.25).

Overall, introducing unambiguous discs reduced, rather than increased, the proportion of time all the discs were perceived as the same color, as predicted if ambiguous neural representations do not link with unambiguous representations.

### Experiment 2: Conventional versus patchwork stimulus arrays

**Figure 5.**
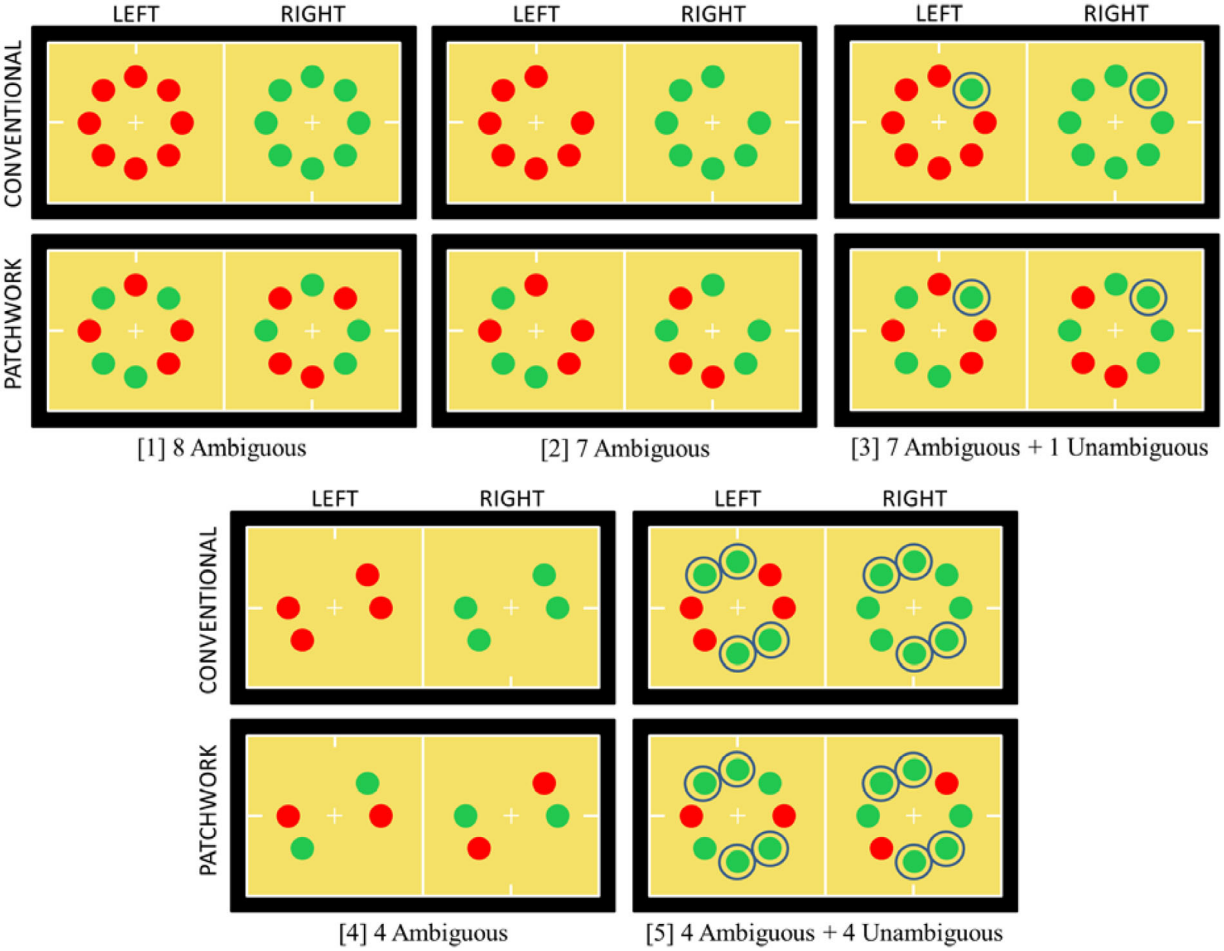
Stimuli in Experiment 2. Conventional arrays of discs, for which the measured percepts are possible by seeing stimuli presented to one eye (first and third rows), and patchwork arrays of discs, for which the measured percepts would not follow from one eye's monocular stimuli alone (second and fourth rows).

Experiment 2 tested if eye-of-origin information influenced the measurements when using CISR in conditions with ambiguous and unambiguous representations together. The aim was to test a result found previously using ambiguous objects alone: Resolution of CISR is explained parsimoniously by a binocular rather than monocular (eye-dominance) neural mechanism (Slezak & Shevell, 2018; Zhang, Slezak, Wang, & Shevell, 2021). Eye-of-origin information, however, may be a linking cue in some circumstances (Stuit et al., 2011). Experiment 2 replicated results from Experiment 1 using patchwork stimulus arrays that at every moment had some discs of each color in both eyes (Figure 5).

The pattern of chromaticities presented in each monocular image was varied. The stimuli presented to each eye could be in either conventional or patchwork arrays (Kovács et al., 1996) (Figure 5). In conventional arrays, all ambiguous discs in CISR presented to each eye were the same chromaticity at any given moment. In patchwork arrays, half of the discs presented to one eye were “green” and the remaining discs in that eye were “red,” their positions randomized from trial to trial. For example, with eight discs presented with CISR (condition 1 in Figure 5), four “green” discs and four “red” discs were shown to one eye; discs with rivalrous chromaticities were presented at corresponding locations in the other eye. The goal of varying the array type was to test whether there was a monocular eye-of-origin contribution to the resolution of neural ambiguity, specifically when ambiguous and unambiguous objects were intermixed. The measured percepts with all discs seen with the identical color would occur more frequently with conventional than patchwork arrays if eye-of-origin information contributes to perceptual resolution of ambiguity.

Display types from Experiment 1 with eight ambiguous discs (condition 1 in Figure 5), seven ambiguous discs (conditions 2 and 3 in Figure 5), or four ambiguous discs (conditions 4 and 5 in Figure 5) were each tested with the two array types (conventional and patchwork). Runs with one ambiguous disc were excluded because differentiating conventional from patchwork arrays requires at least two ambiguous discs.

**Figure 6.**
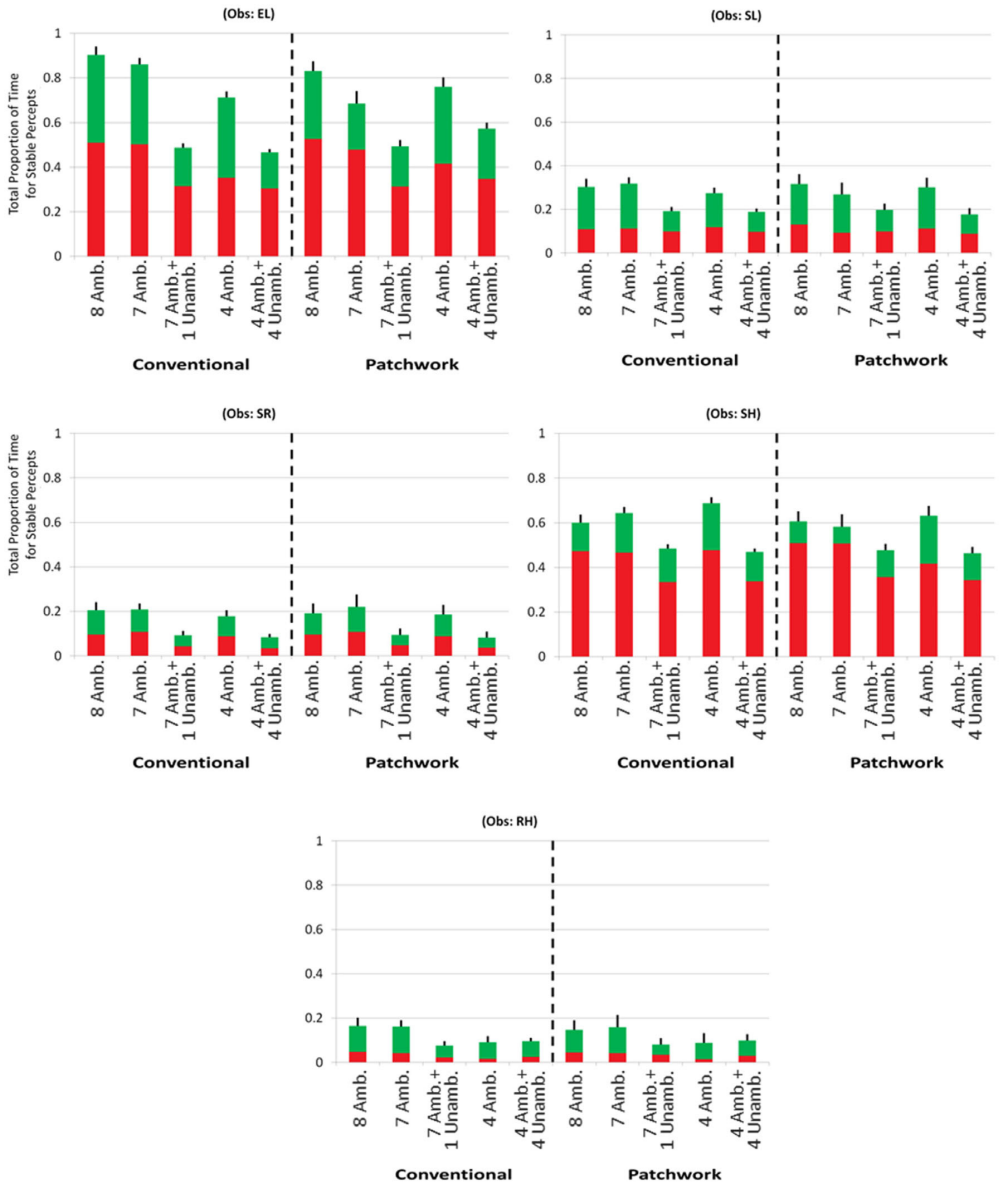
Results from Experiment 2. These results were used to test the hypotheses in Table 1.

Five observers participated in this experiment. Measurements are shown in Figure 6 with each observer's results in a separate panel. Four planned orthogonal contrasts were tested separately for each observer, in addition to testing for an overall difference between conventional versus patchwork arrays (Table 1).

**Table 1.** Weights for planned orthogonal contrasts in Experiment 2.

	Condition					
Array	1	2	3	4	5	Ψ_5_
Conventional	Eight ambiguous	Seven ambiguous	Seven ambiguous + one unambiguous	Four ambiguous	Four ambiguous + four unambiguous	1
Patchwork	Eight ambiguous	Seven ambiguous	Seven ambiguous + one unambiguous	Four ambiguous	Four ambiguous + four unambiguous	−1
Ψ_1_	0.333	0.333	−0.5	0.333	−0.5	
Ψ_2_	0	0	1	0	−1	
Ψ_3_	0	1	0	−1	0	
Ψ_4_	1	−0.5	0	−0.5	0	

Contrasts Ψ_1_ through Ψ_4_ were averaged across the two array types (conventional and patchwork). Ψ_1_ compared whether the total proportion of dominance time was significantly different between the all-ambiguous conditions and the mixed-ambiguity conditions, pooling across the different proportions of ambiguous and unambiguous discs. This contrast was significant for four of the five observers: *p* < 0.05, *t*(10) > 2.38 for all observers except SH, whose measurements were in the same direction. It demonstrated significantly higher dominance times in all-ambiguous conditions compared with mixed-ambiguity conditions. Contrasts Ψ_2_ and Ψ_3_, respectively, were constructed to test whether the number of ambiguous or unambiguous discs presented affected the dominance time for the mixed-ambiguity conditions or the all-ambiguous conditions. Neither contrast reached significance for any observer: For nine of these 10 contrasts, *t*(10) < 0.7 so *p* > 0.5; in one case, *t*(10) < 1.87 so *p* > 0.09. Ψ_4_ was constructed as a control to test for a reduction in dominance time due to presenting fewer than eight ambiguous discs. No observer showed a significant difference for this contrast: *t*(10) < 1.3 so *p* > 0.2 for each of the five observers). Overall, these results were consistent with Experiment 1, which found that including unambiguous discs with ambiguous discs reduced dominance time compared with ambiguous discs seen alone.

Contrast Ψ_5_ tested for a difference between the patchwork and conventional stimulus arrays. The purpose of this contrast was to consider if measurements made using CISR include a monocular eye-of-origin contribution to the resolution of visual ambiguity when mixes of ambiguous and unambiguous discs were presented together. If so, conventional arrays would cause a larger proportion of time with all discs the same color compared with patchwork arrays. No observer showed a significant difference for this contrast: *t*(10) < 1.25, *p* > 0.2 for each of the five observers. This finding indicated that array type (conventional or patchwork) did not have a significant effect on the amount of time seeing all disks of the same color. This is the expected result if there is no contribution from eye-of-origin information to linking ambiguous percepts together.

Regarding the main question of whether ambiguous discs link with unambiguous discs, a further analysis combined results across the observers in Experiment 2. The results corroborated the conclusions found with conventional arrays in Experiment 1 and extended them to patchwork arrays. First consider the conventional stimulus arrays (left half of each observer's panel in Figure 6). When eight disks were in view, the largest proportion was condition 1 with all ambiguous discs; conditions 3 and 5, which also had eight discs but some of them were unambiguous, always were lower. This held for each of the five observers (*p* < 0.01 by a binomial test for five observers, each with a chance probability *p* = 1/3). The same held for comparing condition 2 with seven all-ambiguous discs to conditions 3 and 5. Further, and most importantly here, the same conclusion for condition 1 versus conditions 3 and 5 held for experiments with patchwork arrays (right half of the panel for each observer), showing that adding unambiguous discs reduced dominance time with patchwork as well as conventional arrays (*p* < 0.01); again, this also held for condition 2 with seven ambiguous discs in comparison to conditions 3 and 5 at the same level of significance. In sum, all of these results are consistent with ambiguous neural representations not linking with unambiguous representations.

### Experiment 3: Control for potential residual flicker

The previous experiments support the view that ambiguous objects are not grouped with unambiguous objects. This assumed, however, that CISR discs and unambiguous discs were differentiated based on their ambiguity and not any perceivable residual chromatic flicker, which sometimes is seen using CISR. Even though heterochromatic flicker photometry was used to establish equiluminance for each observer, a small flicker percept may persist.

**Figure 7.**
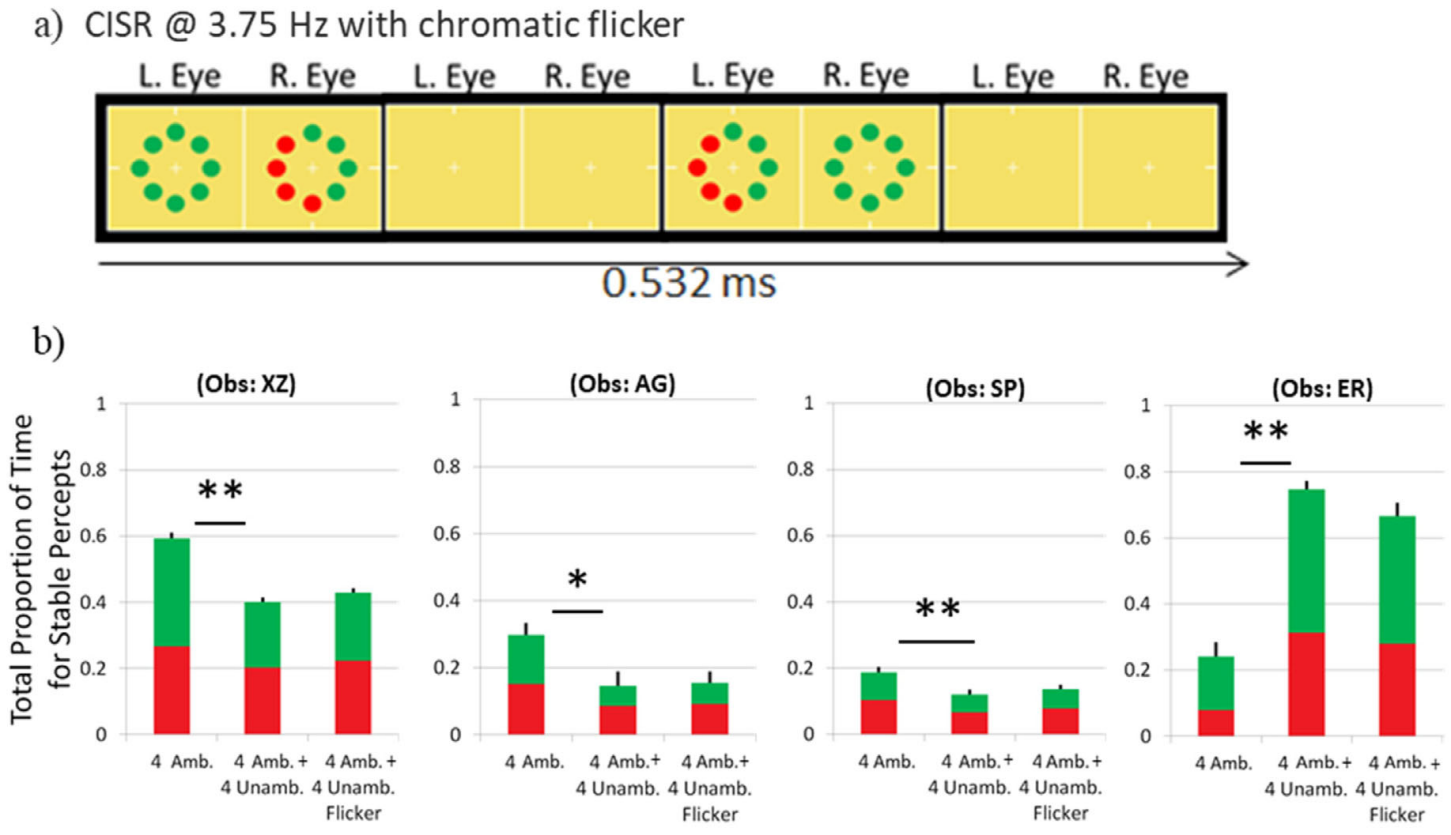
(a) Time course of stimulus display with chromatic flicker intentionally introduced in the unambiguous discs. Note that the ambiguous CISR discs also were turned off at the same times as the unambiguous discs. (b) Results from Experiment 3 testing whether residual perceived flicker may drive separate grouping of ambiguous and unambiguous discs. *p < 0.05, **p < 0.01.

To test whether perceived flicker could be selectively linking the ambiguous stimuli, a new condition introduced flicker for the unambiguous discs by rapidly turning them on and off (i.e., by changing them back and forth to the same chromaticity as the background) in phase with the CISR eye swaps. The unambiguous disc stimuli were on for 133 msec followed by an equal duration with the stimuli off (Figure 7a). The discs continued to cycle on and off for the entire duration of a trial. The CISR discs were also turned on and off to rule out the possibility of the ambiguous and unambiguous discs grouping separately based on periods with only the ambiguous discs in view when the unambiguous discs were not. Three conditions were tested: four ambiguous discs presented alone (all-ambiguous condition, a replication), four ambiguous discs presented together with four unambiguous discs as in the previous experiments (mixed-ambiguity condition, a replication), and the new condition with four ambiguous discs and four unambiguous discs presented together and turned on and off every 133 msec (mixed-ambiguity with flicker condition). A comparison between the last two conditions tested for an effect of residual chromatic flicker. Measurements from each observer are shown in a separate panel in Figure 7b.

For each observer, two non-orthogonal contrasts were planned with Bonferroni-corrected tests. The main comparison of interest between the mixed-ambiguity condition used in earlier experiments and the mixed-ambiguity with flicker condition was never significant for any observer: largest *t*(6) < 1.83 among these observers; moreover, the *t* value was not significant (*p* > 0.10) even as a more powerful planned two-tailed contrast without Bonferroni correction. This was the expected result if possible residual flicker in the ambiguous discs was not the cause of separately grouping ambiguous and unambiguous stimuli.

Replicating results in Experiments 1 and 2, the dominance time for the all-ambiguous condition was significantly greater than for the mixed-ambiguity condition for three of four observers: *t*(6) > 4.1, *p* < 0.01. A fourth observer (ER) showed a lower proportion of time for the all-ambiguous compared with the mixed-ambiguity condition and was the only subject with this result among any of the 13 observers tested across the three experiments.

## Discussion

Can the ambiguity itself implicit in visual neural representations be used as a feature to link the percepts of multiple objects? Identical ambiguous representations from multiple stimuli viewed simultaneously reveal grouping in that alternating percepts from the stimuli tend to change synchronously for all of the objects in view (Adams & Haire, 1958; Ramachandran & Anstis, 1985; Grossman & Dobbins, 2003; Slezak & Shevell, 2018). Using chromatic interocular-switch rivalry, the experiments here tested for linking by ambiguity in common among the representations when an alternate cue from unambiguous percepts also was available to influence perceptual resolution. Grouping was assessed by the amount of time that all presented objects were seen to have the identical appearance (cf. Kovács et al., 1996; Stuit et al., 2011; Dobbins & Grossman, 2018)

Taken together, the experiments support that the ambiguity implicit in a stimulus representation can be a linking feature mediating interocular grouping. Ambiguous representations and unambiguous representations presented together, however, were not found to group with each other. In short, separate representations in the visual field that had identical ambiguity consistently appeared the same color, but their appearance was not found to be attracted to a nearby unambiguous hue.

### What is neural ambiguity?

By definition, something is ambiguous if it is open to more than one meaning or interpretation. For example, an ambiguous sentence has two different possible meanings (e.g., “The doctor saw the patient with one eye”). The ambiguity does not arise from uncertainty about encoding the words in the sentence. Similarly, a Necker cube is ambiguous because it may be interpreted (that is, perceived) in one of two plausible orientations. In fact, any three-dimensional percept of a cube reflects resolution of neural ambiguity because the neural representation of the planar retinal image does not require a three-dimensional interpretation. The lines composing a Necker stimulus are sharp and clear, of course, so the retinal image created by the lines is precisely encoded at the initial level of neural representation. This neural response itself is not in question; its neural ambiguity derives from the capability of the representation to evoke more than one percept. Neural representations (or sentences), therefore, are ambiguous because they have two or more interpretations.

### Relation to color–motion binding errors

Prior research on color–motion feature-binding errors found that peripheral percepts are altered by what is seen in the central part of the visual field, at the expense of wrongly portraying the true color–motion conjunction of features in peripheral regions (Wu et al., 2004; Wang & Shevell, 2014; Shevell & Wang, 2016). In this case, the central–field feature conjunction (say, red objects moving upward) spreads to stimuli in the peripheral area, perceptually dominating the true peripheral stimulus conjunction of red objects actually moving downward. The richer neural representation in the central field is thought to dominate the more sparse representation in the periphery, suggesting an analog to the experiments here assuming a peripheral representation is more ambiguous (due to it being less richly represented) than a central–field representation. In experiments here, however, adding an unambiguous color cue to the ambiguous representations did not dominate the percept of the color of the ambiguous objects. Even in the extreme case of a single ambiguous disc presented with seven identical unambiguous chromatic discs (Experiment 1; see Figure 4), there was no evidence that the color of the single ambiguous disc was drawn to the unambiguous color seen simultaneously.

Note that the results here are not in conflict with prior studies on color–motion feature-binding errors. An important difference in the work on color–motion feature conjunction is the large visual field composed of a central region 14° wide and a peripheral area on either side of the central region (7° wide flanks located 7°–14° away from fixation). The experiments in the current study, on the other hand, carefully equated the distance from central fixation for all ambiguous and unambiguous stimuli, which were presented within 1.5° of fixation so in a much smaller retinal area than used in color–motion feature-binding studies. One possibility is that the visual system takes accounts of cortical magnification in resolving ambiguity when different stimuli are at quite different distances from the fovea, as in the work on color–motion binding, but this was not a factor in experiments here that found that representations with a common degree of ambiguity were linked with each other rather than with an unambiguous hue.

### Are ambiguous features linked prior to perceptual resolution?

Linking multiple objects by their common ambiguity also implies that these objects are linked without the need to resolve the perceived color of each object. This allows a linking mechanism to exploit more information from these stimuli than from a typical (non-rivalrous) linking feature because linking by two identical chromaticities (both of the rivalrous ones) is more specific than linking by a single chromaticity alone (assuming the base rate of each chromaticity is equal). Ambiguity itself, therefore, has the potential to strengthen the link between objects that have identical ambiguous features while also enhancing the specificity of the link to those objects that share both of the chromaticities competing for perceptual dominance.

Linking multiple objects before disambiguation of their percept suggests that grouping may occur early in visual processing. This is in accordance with neurophysiological evidence that reveals grouping as early as V1 (Sugita, 1999) and consistent with the view that grouping can act at different levels of the visual pathway (Palmer, Brooks, & Nelson, 2003). Multi-level grouping processes could act on both pre- and post-disambiguation object representations.

Barlow's (1981) linking-features hypothesis was proposed as a mechanism for grouping disparate but related parts of a scene “linked” by the strength and number of common features. Typical examples of linking features include color, shape, orientation, and velocity of motion. Common ambiguity may similarly serve as a linking feature between disparate objects. One function of linking features could be to separate a figure from ground so that subsequent processes can act on retinotopically non-contiguous fragments that represent parts of a single object. Representations in various retinal regions that share common ambiguity plausibly may be inferred to share also the same underlying cause for their ambiguity and thus may be linked together. This is similar in principle to shared values of more typical linking features, such as orientation and motion, which can indicate retinal fragments generated by the same object (Beck, 1966; Singh & Fulvio, 2007; Claessens & Wagemans, 2008).

### Unambiguous context with ambiguity mediating three-dimensional percepts

Unambiguous percepts can affect the perceptual resolution of neural representations for motion or form when the stimuli are seen as three-dimensional objects (Grossmann & Dobbins, 2003; Freeman & Driver, 2006; Sundareswara & Schrater, 2008; Klink et al., 2009; Ouhnana & Kingdom, 2016). In this case, unambiguous context can alter the perception of a nearby ambiguous neural representation. Context perceived as three dimensional, which is unlike the context in experiments reported here, can bias perceptual resolution of the complete three-dimensional percept from both ambiguous and unambiguous representations. For example, with two rotating structure-from-motion dot cylinders, one ambiguous and the other unambiguous in its rotational direction, the direction of the ambiguous cylinder follows the unambiguous cylinder but only if they are coaxial. This coupling drops to chance when both cylinders are turned 90° so each has its own rotational axis (Klink et al., 2009). With coaxial rotation the objects can be seen as parts of a single three-dimensional element, perhaps due to prior experience.

Similarly with static objects, such as multiple Necker cubes, an unambiguous representation may drive perception of an ambiguous representation. Three-dimensional unambiguous context can imply a particular viewpoint (either looking down or looking up at the objects) (Sundareswara & Schrater, 2008). When nearby unambiguous cubes are oriented such that an observer would be looking down at them, an ambiguously oriented cube may follow the same viewpoint due to the low likelihood of different views seen simultaneously. Thus, experiments with three-dimensional percepts provide a special case of resolving ambiguity in which unambiguous cues presented simultaneously can constrain a coherent three-dimensional interpretation.

Experiments here, on the other hand, are consistent with ambiguity itself serving as a cue that links the resolution of percepts without an influence from unambiguous chromatic context. This is not in conflict, of course, with previous work using stimuli seen as three-dimensional. Linking based on ambiguity itself could occur at an earlier stage of cortical processing (Polonsky, Blake, Braun, & Heeger, 2000; Kim, Hong, Shevell, & Shim, 2020), whereas three-dimensional contextual effects, which perhaps draw on experience-based plausibility, may emerge at a higher level of neural representation (Sereno, Trinath, Augath, & Logothetis, 2002; Brascamp, Kanai, Walsh, & van Ee, 2010). Ambiguity itself may even link objects with ambiguous three-dimensional form or motion early in visual processing, with that link overridden at a higher neural level based on accrued experience with three-dimensional space, including constraints imposed by an unambiguous three-dimensional context.

## Conclusions

Experiments here were consistent with common ambiguity serving as a feature by which multiple stimuli can be interocularly grouped together, similar to the way that common orientation or motion can evoke linking (Barlow, 1981; Wu et al., 2004; Stepien & Shevell, 2015). Moreover, introducing an unambiguous color cue did not significantly attract the perceptual resolution of color from ambiguous representations. Overall, the results are in accord with ambiguity in common serving as a linking feature that supports perceptual grouping.
